# Unintended Pregnancy and Associated Factors among Women Who Live in Ilu Gelan District, Western Ethiopia, 2021

**DOI:** 10.1155/2022/8646724

**Published:** 2022-01-21

**Authors:** Ephrem Yohannes, Bikila Balis

**Affiliations:** ^1^College of Medicine and Health Sciences, Department of Midwifery, Ambo University, Ambo, Ethiopia; ^2^College of Health and Medical Sciences, Department of Midwifery, Haramaya University, Ambo, Ethiopia

## Abstract

**Background:**

The World Health Organization Report noted that unintended pregnancy is the most common cause of maternal mortality in developing countries. Most unintended pregnancies occur where access to maternal care is limited, and because of this, many mothers lose their life. Therefore, this study was an attempt to assess the proportion of unintended pregnancy and associated factors among women who live in Ilu Gelan District, Western Ethiopia, 2021.

**Method:**

A community-based cross-sectional study was conducted in the rural and urban kebeles of Ilu Gelan Woreda West Shoa Zone, Ethiopia, from March 1 to 30, 2021. The study population consists of 540 pregnant women who were living in Ilu Gelan Woreda for at least the last six months during the data collection period. Simple random sampling by lottery method was used to recruit the study subject. Data were checked, coded, entered to EpiData version 3, and then exported to SPSS version 25 for analysis. Both descriptive and analytical statistical procedures were utilized. Both bivariable and multivariable logistic regressions was implemented.

**Result:**

In this study, the proportion of unintended pregnancy was found to be 55%, at 95% CI: 50.7-59.3. Multivariable logistic regression results showed that married women (AOR = 0.117, CI: 0.04-0.38), monthly income less than 1000 Ethiopian Birr (AOR = 4.93, CI: 1.72-14.09), gravidity greater than or equal to five (AOR = 6.07, CI: 2.4-15.28), birth interval less than 2 years (AOR = 3.35 (1.44-7.8)), lack of awareness about contraceptive (AOR = 2.06 (1.03-4.15)), and husband decision-making on health care (AOR = 11.1 (2.07-59.51)) were significantly associated with unintended pregnancy. *Conclusion and Recommendation*. This study indicated that more than half of pregnant women reported that their current pregnancy was found to be unintended pregnancy. Married women, family monthly income less than 1000 Ethiopian Birr, gravidity greater than or equal to five, birth space less than two years, lack of contraceptive awareness, and health care decisions by husband only showed a significant association with unintended pregnancy. To decrease the current level of unintended pregnancy, all concerned stakeholders should emphatically consider those identified factors for intervention; specifically, Ilu Gelan District Health Bureau and health providers should empower women with health education about family planning and decision-making related to their health issues in the study area.

## 1. Introduction

Pregnancy is a happy event for the woman, husband, family, and community when it is wanted or intended. However, millions of pregnancies in the world ended to be unintended due to many factors [1]. Unintended pregnancy is a pregnancy that occurred when no children were desired or earlier than the desired point of time [2]. Over 100 million acts of sexual intercourse take place each day in the world, resulting in around 1 million conceptions, about 50% of which are endeavour unplanned and about 25% are definitely unwanted pregnancies [3].

The complications and risks of unprotected sex, unintended pregnancy, and sexually transmitted disease are extremely connected to unintended pregnancy [4]. The study indicated that women with a history of intimate partner violence (IPV) had significantly higher likelihoods of unintended pregnancy, and reducing IPV by 50% could possibly decrease unintended pregnancy by 2%-8% [5]. Unsafe abortion, caused by unintended pregnancies, signifies a significant public health task all over the world. Although numerous approaches have been used to report this problem, unsafe abortion from unwanted pregnancy continued to be the reason for a significant figure of maternal morbidity and mortality [6]. The fundamental public goal is to avert unplanned pregnancies and empowering women to plan their period of pregnancy. Not only this but also, unplanned pregnancy has relation with basic demographic patterns to women's educational achievement and to families' financial welfare [7].

Globally, it was estimated that there are 87 million cases of unintended pregnancies, and 46 million cases resulted to be induced abortion. More than 18 million of 46 million pregnant women end up with unsafe abortions [8]. Unintended pregnancy can be prevented through higher coverage of sexual and reproductive health services [9]. Even though the rate of unintended pregnancy has decreased in both developed and undeveloped countries, the problem is not resolved yet [10, 11]. Worldwide, 44% of pregnancies were unintended in 2014 [9]. While the fall in unintended pregnancy rates in developed regions matched with a reduction in abortion rates, the decline in low-income countries concurred with a fall in ectopic pregnancy rates. In 2010-14, 59% of unintended pregnancies ended in abortion in developed regions, whereas 55% of unintended pregnancies happened in developing countries [9, 12].

The problem of unintended pregnancy affects an enormous proportion of society. The growing plea for lesser families, reducing the age at first sex, and raising the age of marriage have meant that many women expend much of their adult lives trying to escape an unintended pregnancy [13]. Unintended pregnancies can have contrary physical, mental, social, and economic consequences. Mainly, unlawful abortions and related difficulties often follow unintended pregnancies and affect the lives of many women in developing countries [14]. Unintended pregnancy is one of the most community health issues in the world, and it is the main sexual and reproductive health difficulty that brings sophisticated jeopardy of morbidity and mortality for women, often due to unsafe abortion [15].

Few investigators in Ethiopia have reported the concern associated with unintended pregnancy, and little has been discussed about its cause particularly in the rural area. Moreover, determinations to decrease the occurrence of unintended pregnancy were very fragile. In Ethiopia, the required fertility rate is 3.6 offspring as compared with the actual total fertility rate, which is 4.6 children. This shows that the women in Ethiopia are at the problem of having, on average, one child more than they want to have. According to the report of the Ethiopian Demographic Health Survey, the prevalence of unintended pregnancy in Ethiopia is 25%, of which 17% are unplanned and 8% are unwanted [16]. Unintended pregnancy was found to be a main reproductive health problem in Wolaita Zone, Southern Ethiopia [17].

Even though some studies have been done on the prevalence and associated factors of unintended pregnancy in Ethiopia and study area, but there is no community-based study conducted at the study area yet. Therefore, this study was aimed at determining the proportion and the factors associated with unintended pregnancy among pregnant mothers attending ANC at Ilu Gelan district, West Shoa Zone, Oromia region, Ethiopia.

## 2. Methods

### 2.1. Study Setting

This community-based cross-sectional study was conducted in the Ilu Gelan district selected kebeles from March 1 to 30, 2021. Ilu Gelan district is found in West Shewa Zone, Oromia regional state, and 221 km away from Addis Ababa to Western Ethiopia. The district has 28 kebeles (27 rural and 1 urban). The total population of the district is 88,045; the reproductive age group is 19,484; and the number of pregnant women is 3,055. There are three health centres, 17 health posts, and 1 government hospital in the district [18].

### 2.2. Study Population and Study Design

A community-based cross-sectional study was conducted from March 1 to 30, 2021, among pregnant women who lived in Ilu Gelan Woreda at least for the last 6 months.

All the pregnant women who were living in the Woreda during the data collection period were included, but the ones who were unable to communicate and seriously ill women were excluded from the study.

### 2.3. Sample Size Determination and Sampling Technique

The total sample size used for this study was determined using a single population proportion formula. By considering the proportion of unintended pregnancy, which was taken from the study done in Ethiopia, *p* = 33.5 % [19], 95% confidence level, *z* and a margin of error 5%, the final sample size after considering 10% nonresponse and 1.5 design effect was 568 study participants. A multistage stratified sampling technique was used to select the participants. The information received from the Woreda Health Office indicated that there are averages of 100 pregnant women in each kebele (the smallest kebele). The total 28 kebeles in the district have been stratified into two (27 rural and 1 urban kebeles). Finally, eight rural kebeles and one urban kebele were selected by a simple random sampling technique using the lottery method. The final sample size was proportionally allocated to each selected kebele according to the number of pregnant women present in each kebele. Health Extension Workers (HEW) provided a list of pregnant women working in the area, and pregnant women were selected through a simple random sampling technique using computer-generated random numbers.

### 2.4. Operational Definition

Unintended pregnancy is defined as a pregnancy that is either unwanted, such as the pregnancy that occurred when no children or no more children were desired or the pregnancy is mistimed, such as the pregnancy occurred earlier than desired a pregnancy that was either unwanted or mistimed [20].

### 2.5. Data Collection Tools and Procedure

Data were collected through face-to-face interviews using a structured and pretested questionnaire adapted from a different study conducted on the same topic (49, 51). Ten (10) trained BSc midwives collected the data, and the data collection were supervised by 2 masters in public health holders. The questionnaire was first prepared in English and then translated into the local language Afaan Oromoo and then translated back to English by a third party to check its consistency and conceptual equivalence. The data were properly coded, categorized, and checked for completeness, accuracy, clarity, and consistency by the principal investigator and supervisors before being entered into software for final analysis.

### 2.6. Data Processing and Analysis

The collected data were coded, cleaned, and entered into EpiData version 3.3.1 by two data clerks to minimize logical errors and design skipping patterns. Then, the data were exported to SPSS Window version 25 for analyses. Frequency and crosstabulation were employed to check for any missing values. Descriptive statistics were used to present the proportion of unintended pregnancy, and a binary logistic regression was used to see the association between the outcome variable and each independent variable. The variables that showed *p* ≤ 0.25 in the bivariable analysis were considered as a candidate for multivariable logistic regression analysis (using enter method); this was to control possible confounders and to detect significantly associated factors with unintended pregnancy. Hosmer and Lemeshow goodness of-fit test (0.95) was used to evaluate whether the required assumptions were fulfilled or not. A multicollinearity test was implemented to check the correlation among the independent variables; the test indicated that there was no multicollinearity (VIF < 2). The direction and strength of statistical association were assessed by an odds ratio with 95% CI. The adjusted odds ratio along with 95% CI was computed to identify the associated factors for unintended pregnancy. Finally, statistical significance was declared at *p* < 0.05.

### 2.7. Data Quality Assurance

Data were collected through a pretested structured face-to-face interview questionnaire. Additionally, 1-day training was provided for data collectors and supervisors by the principal investigator to create awareness on the data collection time, the basic technique of data collection, approaches, and the issue of confidentiality and privacy. To get informed consent and reliable data, a clear explanation of the purpose and procedure of the study was given to the study participants. Moreover, supervisors supervised the data collectors daily. The supervisors and principal investigator for completeness and consistency checked the filled questionnaire daily.

## 3. Result

### 3.1. Sociodemographic Characteristics

In this study, we interviewed 540 women who were living in the Ilu Gelan District, yielding a response rate of 95%. The mean age of the study participants was 29.30 (SD ± 4.577), and 194 (35.9%) participants were within the age group of 30-34 years. Out of the 540 respondents, 328 (60.7%) were urban dwellers, 328 (60.7%) were from the Orthodox religion. More than two-thirds of the respondents were from the Oromo ethnic group. Most of the respondents were only able to read and write 216 (40.0%). Moreover, most women 222 (41.1%) were housewives, and half of them were married, and most of their husbands, 236 (43.7%), were farmers. Nearly half of the respondents 260 (48.1%) were living with greater than six family members (Table 1).

### 3.2. Pregnancy and Health-Related Characteristics

Most of the respondents had greater than five gravidity 235 (43.5%), and nearly half of participants 243 (45%) were multiparous mothers. Majority, 204 (37.8%), of the respondents gave birth in less than two-years birth intervals. Most of the respondents194 (35.9%) have a son baby. Nearly two-thirds of the respondents 388 (71.9%) have no awareness of ovulation time. Not only this but also, the majority of the respondents 347 (64.3%) have no ANC visits (Table 2).

### 3.3. Prevalence of Unintended Pregnancy

**Figure 1:**
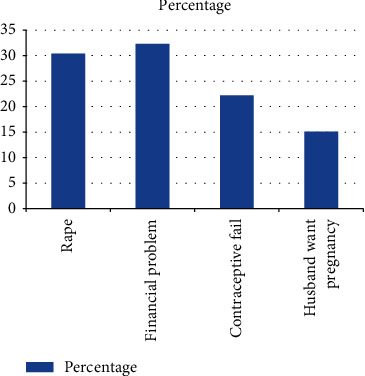
The reason of unintended pregnancy among pregnant women living in Ilu Gelan district, West Shewa Zone, Ethiopia, 2021 (*n* = 540).

**Figure 2:**
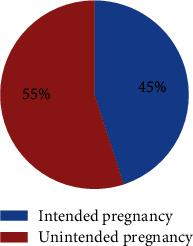
Proportion of unintended pregnancy among pregnant women living in Ilu Gelan district, West Shewa Zone, Ethiopia, 2021 (*n* = 540).

The majority of the respondents, 302 (55.9%), have history of unintended pregnancy. Most of the respondents 97 (32.7%) did not want the current pregnancy because of their financial problems to raise the child (Figure 1). More than half of the respondent's current pregnancy 297 (55%) were unintended pregnancy (Figure 2).

### 3.4. Contraceptive Method Awareness

Majority of the respondents, 301 (55.7%), heard about contraceptive methods. The source of information for most participants 106 (35.2%) was media. Most of the respondents 168 (31.1%) have awareness of condoms as a modern contraceptive. More than half of the respondents 304 (56.3%) have communicated about contraceptive methods with their spouse. Majority of the participants 120 (60.3%) ever used pills as a modern contraceptive method. The health care autonomy (all health-related services) was majorly 266 (49.3%) decided jointly with their husbands. In addition to this, the majority, 234 (43.3%), said that their family planning utilization was decided by their husbands. Not only this but also, the majority's 243 (45%) family income was decided by their husbands. The majority of the respondents, 360 (66.7%), traveled more than two hours to arrive at health institutions (Table 3).

### 3.5. Factors Associated with Unintended Pregnancy

Multiple logistic regression analysis indicated that married pregnant women who had husbands were 11% less likely to experience unintended pregnancy than widowed women (AOR = 0.117, CI: 0.04-0.38). The pregnant women whose monthly income was less than 1000 Ethiopian Birr were (AOR = 4.93, CI: 1.72-14.09) almost 5 times more likely to report their pregnancy was unintended than pregnant women who have monthly income of greater than 10,000 Ethiopian Birr. The women who had gravidity greater than or equal to 5 were 6 times more likely to report their pregnancy was unintended (AOR = 6.07, CI: 2.4-15.28), than those who had gravidity of less than or equal to two. The pregnant women who have less than two years of birth interval were (AOR = 3.35 (1.44-7.8)) three times more likely to experience unintended pregnancy than those who have a birth interval greater than or equal to 2 years. The pregnant women who have no awareness about contraceptives were (AOR = 2.06 (1.03-4.15)) two times more likely to have an unintended pregnancy than those pregnant women who have awareness about contraceptives. The pregnant women whom their husband or partner decided about their health care were (AOR = 11.1 (2.07-59.51)) eleven times more likely to experience unintended pregnancy than those who decided their health care by themselves (Table 4).

## 4. Discussion

This study showed that the prevalence of unintended pregnancy among pregnant women who live in the Ilu Gelan district was 55% (95% CI: 50.7-59.3). The factors significantly associated with unintended pregnancy were marital status, monthly income, gravidity, birth space, awareness of contraceptive, and health care decision.

The prevalence of unintended pregnancy found in this study is in line with a study conducted in Nepal [21] which was 54.5%. In addition to this, a study conducted in Tanzania showed a high prevalence (47.5%) of unintended pregnancy [22]. The study conducted in Southern Ethiopia and Botswana revealed that the prevalence of unintended pregnancy was found to be nearly 43 percent among married women of reproductive age [23, 24]. However, a study conducted in South Africa (64%) indicated that the prevalence of unintended pregnancy was higher than that of the current study [25]. Most of the studies from different countries (Sudan Khartoum 30%, India 22%, Addis Ababa 28%, Oromia regional state 39.8%, Northern Ethiopia 20.6%, and Southern Ethiopia 36.8%) showed that the prevalence of unintended pregnancy was found to be lower than the current study [1, 26–30]. The reason why most of the studies reported lower than the current study was that most of the studies were conducted at the health institution level. The other reason may be due to differences in sample size, study population, and sociocultural characteristics and, as well as, due to geographical barriers across the countries.

The current study showed that married pregnant women who had husbands were eleven percent less likely to experience unintended pregnancy than widowed women. In line with the current finding, a community-based cross-sectional study conducted in Ethiopia revealed that being formerly married and separated and never married was significantly associated with unintended pregnancy [31]. The same as the current study in Oromia region Gelemso town being divorced/widowed marital statuses was significantly associated with unintended pregnancy than married women [32]. The reason behind this might be that a widowed woman has a higher chance to have sex with different persons. The other possible reason could be due to economic problems. This can increase the chance of having unintended pregnancies than those married women.

This study indicated that unintended pregnancy was five times higher among women who have a monthly income of less than a thousand Ethiopian Birr than those earning more than ten thousand Ethiopian Birr. This finding is similar to a study conducted in Southern Ethiopia [17]. The survey conducted by the WHO showed that the burden of unintended pregnancy disproportionately affects the poor [11]. A systematic and meta-analysis observational study conducted in Ethiopia revealed that unintended pregnancy was common among low-income pregnant women [26]. This implies that it is hard to feed their baby after giving birth so that the women did not want the unplanned pregnancy.

Grand multigravida women (gravid more than equal to five) experienced unintended pregnancy more likely than pregnant women with gravida of less than two. This finding is supported by evidence obtained from the systematic review conducted on twenty-two papers, which indicated that unintended pregnancy was common among multigravid women [33]. The study conducted in Tanzania also showed that there was an association between unintended pregnancy and grand multigravida women [22]. Not only this but also, a study done in Arsi Negele, Ethiopia, showed that grand multigravida women experienced unintended pregnancy than women with gravida of two [34]. In addition to this, a study conducted in Hossana, Ethiopia, showed that multigravida women had experienced unintended pregnancy than others [35]. In Southern Ethiopia, researchers explored that women with lower parity are less risky to have unintended pregnancy than high parity mothers [23]. Moreover, the community-based cross-sectional study conducted in Ethiopia revealed that being gravidity > 5 is significantly associated with unintended pregnancy [31]. This is because high-gravidity women might already have adequate children and practice sex for enjoyment rather than to have children. In addition, the reason why they are grand multigravida may be due to unplanned pregnancy. These findings can be explained by the fact that couples from rural areas prefer to have more sons and they may end up having more children.

Pregnant women with a birth interval of fewer than two years were more likely found to have unintended pregnancy. This finding is similar to a study conducted in Pakistan that indicated birth spacing less than one year was strongly associated with the unintended pregnancy [36]. The cross-sectional study conducted at Yumbe Hospital showed that unplanned pregnancy was significantly associated with a short birth interval [37]. Limited access to reproductive health services in some settings may lead to unwanted or unplanned pregnancies for those women who wish to use contraceptives at present or in the future, leading to the notion of unmet need for family planning and therefore short birth intervals [38]. Similar to parity, short birth intervals of less than 12 months were also found to be significantly associated with unintended pregnancies in our study as has been noted elsewhere [39]. A study conducted at the University of Cincinnati Medical Centre showed that short birth spacing is significantly associated with unintended pregnancy [40]. This could probably be because women/couples who plan their pregnancy may follow the recommendations for child spacing and therefore end up with optimal birth intervals.

The current study indicated that the pregnant women who have no awareness about contraceptives were two times more likely to have an unintended pregnancy than those pregnant women who have awareness about contraceptives. Consistent with the available literature [22, 41–43], our study found that unintended pregnancy is strongly associated with a lack of awareness of contraceptive methods. In line with this finding, a study conducted in South-Western Ethiopia showed that unintended pregnancy was common among women who have no awareness about contraceptives [2]. The study conducted in the Oromia region Gelemso town showed that having no awareness of contraception was significantly associated with unintended pregnancy [32]. Knowledge about contraceptives was reported to be negatively associated with unintended pregnancy [33]. The reason why this study found a strong positive association between users of modern contraceptives and unintended pregnancy can be assumed that method failure might have increased the chances of unintended pregnancy among these women.

The other strong evidence this study found out is that pregnant women whose husband or partner decided about their health care were eleven times more likely to experience unintended pregnancy than those who decided their health care by themselves. Similar to this finding, a study conducted in Bale showed that their husband's decision-making style in the household significantly increases the chance of unintended pregnancy [44]. In Southern Ethiopia's study, it showed that unintended pregnancy was less likely to happen among pregnant who have autonomy in their health care [23]. A study done in Bangladesh stated that a unit increase in the autonomy scale of women decreases the odds of unintended pregnancy by sixteen percent [45]. A study conducted among pregnant Nepalese women found a significant relationship between women's autonomy and unintended pregnancy in a bivariate analysis [46]. A study conducted among pregnant women in Northwest Ethiopia and Arsi Negele revealed that making family planning decisions on their own was preventive against unintended pregnancies [14, 31]. The strength of this study was that it was community-based; it might indicate the true rate of unintended pregnancies in the community, as many of the clients with unintended pregnancy are less likely to visit health institutions. However, the responses of the respondents might be liable to social desirability bias specifically underreporting of unintended pregnancy due to domination and fear of their husband and community around them.

## 5. Conclusion and Recommendation

The prevalence of unintended pregnancy among the pregnant women living in the Ilu Gelan district was found to be high. This study identified widowed marital status, low family monthly income, grand multigravidas, birth interval less than two years, lack of awareness about contraceptive, and health care autonomy decided by their husband as factors significantly associated with unintended pregnancy.

All concerned bodies should pay particular attention to the factors that directly limit the decision-making authority of women in Ethiopia. The West Shewa Zone Health Office should provide contraceptive services especially the modern methods to women during preconception and postpartum. In addition, the health care providers need to give health education on the advantage of autonomic decisions of women and effective utilization of contraceptives, as it minimizes unintended pregnancy. Finally, we would like to recommend researchers to conduct further mixed qualitative and quantitative studies in the study area.

## Figures and Tables

**Table 1:** Sociodemographic characteristics of respondents among pregnant women living Ilu Gelan district, West Shewa Zone, Ethiopia 2021 (*n* = 540).

Variables	Category	Frequency	Percent
Age	20-24	70	13.0
	25-29	193	35.7
	30-34	194	35.9
	>35	83	15.4

Residence	Urban	212	39.3
	Rural	328	60.7

Religious	Protestant	212	39.3
	Orthodox	328	60.7
	Muslim	77	14.3
	Others^∗^	61	11.3

Ethnicity	Oromo	411	76.1
	Amhara	78	14.4
	Gurage	29	5.4
	Others^∗∗^	22	4.1

Educational level	Unable to read and write	178	33.0
	Only read and write	216	40.0
	Primary school [1–8]	42	7.8
	Secondary [9–12]	56	10.4
	College and above	48	8.9

Marital status	Single	92	17.0
	Married	270	50.0
	Divorced	132	24.4
	Widowed	46	8.5

Mother's occupation	Government employee	95	17.6
	Merchant	125	23.1
	Student	38	7.0
	Housewife	222	41.1
	Farmer	45	8.3
	Daily labor	15	2.8

Husband occupation	Government	147	27.2
	Merchant	123	22.8
	Student	34	6.3
	Farmer	236	43.7

Family size	≤2	105	19.4
	2-5	175	32.4
	≥6	260	48.1

Monthly income	<1000	51	9.4
	1000-10000	399	73.9
	≥10,000	90	16.7

Distance	<2 hours	180	33.3
	≥2 hours	360	66.7

^∗^Waqeffata, Catholic, and Atheist; ^∗∗^Gambella, Afar, and Tigre.

**Table 2:** Pregnancy and health-related characteristics of pregnant women living in Ilu Gelan district, West Shewa Zone, Ethiopia, 2021 (*n* = 540).

Variables	Category	Frequency	Percent
Gravidity	1-2	241	44.6
	3-4	129	23.9
	≥5	170	31.5

Parity	Nulliparous	201	37.2
	Multiparous	243	45.0
	Grand multiparous	96	17.8

Birth interval	Nulliparous	201	37.2
	<2	204	37.8
	≥2	135	25.0

Having son	Nulliparous	201	37.2
	Yes	194	35.9
	No	145	26.9

Ovulation time knowledge	Do not know	388	71.9
	Know	152	28.1

ANC visit	Yes	347	64.3
	No	193	35.7

History of unintended pregnancy	No	238	44.1
	Yes	302	55.9

**Table 3:** Awareness towards contraceptive method of pregnant women living in Ilu Gelan district, West Shewa Zone, Ethiopia, 2021 (*n* = 540).

Variables	Category	Frequency	Percent
Do you know contraceptive method?	No	239	44.3
	Yes	301	55.7

Source of information	Media	106	35.2
	Health professionals	65	21.6
	Husband	78	25.9
	Peer	52	17.2

Which one of the following modern contraceptives do you know?	Pills	125	23.1
	Condom	168	31.1
	Injectable	120	22.2
	Implants	128	23.7
	IUCD	136	25.2
	Female sterilization	87	16.1
	Male sterilization	56	10.4

Spousal communication about contraceptives	No	236	43.7
	Yes	304	56.3

Ever used contraceptive	No	341	63.1
	Yes	199	36.9

Type of contraceptive used	Pills	120	60.3
	Condom	100	50.3
	Injectable	104	52.3
	Implants	95	47.7
	IUCD	86	43.2

Prevention of unintended pregnancy awareness	No	285	52.8
	Yes	255	47.2

Do you know of emergency contraceptive	No	362	67.0
	Yes	178	33.0

Health care decision (all health services)	Husband or partner	140	25.9
	Me and husband jointly	266	49.3
	Me	134	24.8

Family planning use decision	Husband or partner	234	43.3
	Me and husband jointly	184	34.1
	Me	122	22.6

Family income decision	Husband or partner	243	45.0
	Me and husband jointly	181	33.5
	Me	116	21.5

Means of communication	Radio	71	13.1
	Television	228	42.2
	Others_∗_	22	4.1
	None	106	19.6

^∗^Internet, telephone, and cell phone.

**Table 4:** Bivariate and multivariate logistic regression of factors of unintended pregnancy among pregnant women living in Ilu Gelan district, West Shewa Zone, Ethiopia, 2021 (*n* = 540).

Variables	Category	Unintended pregnancy				COR (95% CI)	AOR (95% CI)	*p* value
		Yes		No				
		No	%	No	%			
Age	20-24	44	62.9	26	37.1	1.00	1.00	
	25-29	107	55.4	86	44.6	2.697 (1.4-5.19)	0.99 (0.31-3.21)	
	30-34	114	58.8	80	41.2	1.983 (1.17-3.35)	0.73 (0.19-2.68)	
	>35	32	38.6	51	61.4	2.271 (1.34-3.84)	0.35 (0.08-1.56)	

Educational level	Unable to read and write	101	56.7	77	43.3	1.02 (0.54-1.94)	0.34 (0.11-1.13)	
	Only read and write	126	58.3	90	41.7	1.08 (0.56-2.05)	0.51 (0.15-1.62)	
	Primary school	20	47.6	22	52.4	0.70 (0.30-1.62)	0.65 (0.15-2.76)	
	Secondary	23	41.1	33	58.9	0.54 (0.25-1.18)	0.23 (0.06-0.98)	
	College and above	27	56.3	21	43.8	1.00	1.00	

Marital status	Single	67	72.8	25	27.2	1.17 (0.54-2.55)	3.22 (0.49-21.05)	
	Married	113	41.9	157	58.1	0.32 (0.16-0.6)	0.117 (0.04-0.38)^∗^	0.01
	Divorced	85	64.4	47	35.6	0.79 (0.38-1.6)	0.58 (0.17-1.98)	
	Widowed	32	69.6	14	30.4	1.00	1.00	

Husband occupation	Government	86	58.5	61	41.5	1.00	1.00	
	Merchant	76	61.8	47	38.2	1.147 (0.70-1.87)	1.57 (0.58-4.28)	
	Student	11	32.4	23	67.6	0.34 (0.15-0.74)	1.76 (0.46-6.72)	
	Farmer	124	52.5	112	47.5	0.783 (0.52-1.19)	1.35 (0.57-3.19)	

Family size	≤2	74	70.5	31	29.5	2.38 (1.47-3.87)	2.13 (0.73-6.16)	
	2-5	93	53.1	82	46.9	1.134 (0.773-1.65)	0.74 (0.35-1.56)	
	≥6	130	50	130	50	1.00	1.00	

Monthly income	<1000	31	60.8	20	39.2	2.39 (1.47-3.87)	4.93 (1.72-14.1)^∗^	0.03
	1000-10000	225	56.4	174	43.6	1.13 (0.77-1.65)	1.13 (0.77-1.65)	
	≥10,000	41	45.6	49	54.4	1.00	1.00	

Gravidity	1-2	136	56.4	105	43.6	1.00	1.00	
	3-4	60	46.5	69	53.5	0.67 (0.44-1.032)	2.23 (0.94-5.33)	
	≥5	101	59.4	69	40.6	1.13 (0.76-1.68)	6.07 (2.4-15.28)^∗^	0.01

Birth interval	<2	110	53.9	94	46.1	1.93 (1.237-3.04)	3.35 (1.44-7.8)^∗^	0.01
	≥2	51	37.8	84	62.2	1.00	1.00	

Having son	Yes	55	37.9	115	59.3	1.00	1.00	
	No	79	40.7	90	62.1	2.38 (1.532-3.70)	1.94 (0.91-4.14)	

ANC visit	Yes	154	44.4	193	55.6	1.00	1.00	
	No	143	74.1	50	25.9	3.58 (2.43-5.27)	0.71 (0.31-1.6)	
History of unintended pregnancy	Yes	232	76.8	70	23.2	8.82 (5.96-13.04)	1.06 (0.51-2.22)	
	No	65	27.3	173	72.7	1.00	1.00	

Contraceptive awareness	Yes	153	50.8	148	49.2	1.00	1.00	
	No	144	60.3	95	39.7	1.46 (1.040-2.07)	2.06 (1.03-4.15)^∗^	0.042

Spousal communication about contraceptive	Yes	156	51.3	148	48.7	1.00	1.00	
	No	141	59.7	95	40.3	1.41 (0.99-1.98)	1.021 (0.48-2.16)	

Ever used contraceptive	Yes	91	45.7	108	54.3	1.00	1.00	
	No	206	60.4	135	39.6	1.81 (1.27-2.58)	0.69 (0.16-2.85)	

Health care decision	Husband or partner	83	59.3	57	40.7	1.96 (1.22-3.18)	11.1 (2.1-59.51)^∗^	0.01
	Me and husband	157	59	109	41	1.95 (1.28-2.96)	3.78 (0.98-14.62)	
	Me	57	42.5	77	57.5	1.00	1.00	

^∗^Variable of *p* value ≤ 0.05 in multiple logistic regression.

## Data Availability

The datasets used and/or analysed during the current study are available from the corresponding author upon request.

## Abbreviations

ANC: Antenatal care
APH: Ante partum hemorrhage
CDC: Center of Disease Control and Prevention
EDHS: Ethiopia Demographic Health Survey
ETB: Ethiopian Birr
GA: Gestational age
MDG: Millennium development goal
MNCH: Maternal, Newborn, and Child Health
SPSS: Statistical Package for Social Science
SVD: Spontaneous vaginal delivery
WHO: World Health Organization.
